# Organic management increases beneficial microorganisms and promotes the stability of microecological networks in tea plantation soil

**DOI:** 10.3389/fmicb.2023.1237842

**Published:** 2023-09-19

**Authors:** Xinhui Huang, Yuting Zheng, Panfeng Li, Jixiao Cui, Peng Sui, Yuanquan Chen, Wangsheng Gao

**Affiliations:** ^1^College of Agronomy and Biotechnology, China Agricultural University, Beijing, China; ^2^Institute of Environment and Sustainable Development in Agriculture, Chinese Academy of Agricultural Sciences, Beijing, China

**Keywords:** tea, organic planting, bacterial community, fungal community, co-occurrence networks

## Abstract

**Introduction:**

Organic agriculture is highly regarded by people for its commitment to health, ecology, care, and fairness. The soil microbial community responds quickly to environmental changes and is a good indicator for evaluating soil microecology. Therefore, from the perspective of soil microbial communities, elucidating the impact of organic management on soil microecology in tea plantations has great significance for improving local tea plantation systems.

**Methods:**

The study collected bulk soil from organic management (OM) and conventional management (CM) tea plantations in Pu'er City, a major tea-producing area in China, and analyzed their species diversity, structural composition, and co-occurrence networks using metagenomics technology.

**Results:**

Compared with CM, the diversity index (Shannon) and evenness index (Heip) of soil fungi increased by 7.38% and 84.2% in OM tea plantations, respectively. The relative abundance of microorganisms related to the nitrogen cycle increased. Specifically, there was a significant increase in *Rhodobiales*, a 2-fold increase in *Nitrospirae*, and approximately 1.95 and 2.03 times increases in unclassified genera within *Betaproteobacteria* and *Deltaproteobacteria*, respectively. The relative abundance of plant residue degradation species, *Gemmatimonadetes, Ascomycota, and Basidiomycota*, increased by 2.8, 1, and 1.4 times, respectively. The OM was conducive to the establishment of collaborative relationships among bacterial species and increased the diversity and complexity of species relationships in fungal communities. The network stability of soil ecosystems was promoted. The organic tea plantations' keystone taxa contained mycorrhizal fungi (*Pezoloma_ericae, Rhizophagus_irregularis, Rhizophagus_clarus*), as well as species involved in soil nitrogen metabolism (*Acidobacteria_bacterium, Acidobacteriia_bacterium_AA117, Sphingomonas_sp._URHD0007, Enhydrobacter_aerosaccus*), pathogen (*Erysiphe_pulchra*), and parasites (*Paramycosporidium saccamoeba*). The partial least squares method (PLS-SEM) indicated that OM affected N-NH${}_{4}^{+}$ negatively, increasing the abundance of fungi, and thereby positively affecting the Shannon index.

**Conclusion:**

In brief, reasonable organic management can improve the diversity of soil microorganisms, increase the relative abundance of beneficial bacteria in tea plantation soil, and promote the stability of the soil microbial ecological network.

## 1. Introduction

As an important cash crop, tea is widely cultivated in developing countries. In 2020, the global tea plantation area reached 5.098 million ha. China is a big tea-producing country. The tea plantation area reached 3.165 million ha, accounting for 62.1% of the total global tea area in 2020. The total annual production was 2.986 million tons, accounting for nearly 50% of the total global tea production (ITC, 2022). The tea plantation ecosystem provides humans with not only tea but also by-products such as fruits, vegetables, medicinal materials, and poultry. It also maintains the Earth's life system by conserving water resources, improving soil, preventing water and soil loss, mitigating natural disasters, regulating climate, purifying the environment, breeding and preserving biodiversity, having social functions such as ornamental field value, and displaying traditional culture, tourism, and recreation (Xue et al., 2013). Therefore, the sustainability of tea plantation ecosystems is of great significance for the global ecological environment and human wellbeing.

Soil is the foundation of agricultural production. Without healthy and fertile soil, food security cannot be guaranteed. As an important part of the soil environment, soil microorganisms play the roles of decomposers, pathogens, and symbionts in the ecological environment and participate in the decomposition of organic matter, the activation of nutrients, the prevention and control of plant diseases, and the maintenance of soil biodiversity. It drives the biogeochemical cycle on Earth (Stott and Taylor, 2016; Essel et al., 2019; Ney et al., 2020). Microorganisms are small in size, abundant in quantity, and highly sensitive to environmental changes (Karimi et al., 2017; Wagg et al., 2019; Bei et al., 2021; Wang C. et al., 2021; Wang et al., 2022). Microorganisms can serve as biological indicators for evaluating soil quality (Karimi et al., 2017).

Tea trees are perennial crops. The soil acidification caused by the accumulation of leaf residue and rhizosphere exudates has a severe impact on the growth of tea trees (Rothenberg et al., 2022). Previous studies have shown that organic management can prevent soil acidification (Yan et al., 2020). Additionally, organic agriculture based on the principles of health, ecology, care, and fairness has gradually become a highly recommended agricultural production mode by protecting the health and welfare of humans and future generations (FiBL and IFOAM, 2022). Therefore, organic management can provide the possibility for the sustainable development of tea plantation ecosystems.

The characteristics of the soil microbial community, as a sensitive indicator of soil quality, have been extensively studied by researchers. However, these research conclusions were inconsistent. For example, the abundance of bacteria and fungi under organic management was higher than that under conventional management (Kundel et al., 2020), and beneficial microorganisms increased (Suyal et al., 2021). Many studies have shown that, compared with conventional management, the microbial diversity of organic management has not changed or decreased (Karanja et al., 2020). The difference in the microbial community and abundance between organic and conventional farming systems changes with the change in land use (arable land, orchards, and grassland), climate, and other factors (Lori et al., 2017), and there is no unified conclusion yet. Therefore, it is necessary to study the impact of organic management on the microbial community of farmland based on specific climates, crop types, and cultivated land types and to elucidate soil microecological status under organic management.

To date, many studies have analyzed the soil microecology of organic tea plantations from the perspective of soil microbial communities, but relatively little research has been conducted in Pu'er City, the world's tea source. Therefore, the investigation of soil microorganisms in organic tea plantations in this region has reference value for the study of soil microecology in global organic tea plantations and also provides a theoretical basis for the improvement of local organic management systems and tea plantation management systems.

Bulk soil samples from organic and conventional tea plantations in Pu'er City were collected to continue exploring soil microecology changes. The soil bacterial and fungal alpha diversity, composition structure, and co-occurrence network of tea plantations under organic and conventional management were studied through metagenomics. Combining previous studies and tea plantation conditions, we hypothesized that (1) organic management will change the microbial community structure and increase the beneficial microorganisms in the tea plantation soil and (2) organic management will improve the complexity of the microbial symbiosis network and help to build a stable micro-ecological network. The research objectives are (1) to study the impact of organic management on the diversity and community structure of bacteria and fungi in tea plantation soil; (2) to reveal soil biomarkers that distinguished between organic and conventional management, microbial symbiotic patterns, and keystones taxa that exerted considerable influence on microbiome structure and function, and (3) to reveal the relationship between organic management and soil environmental factors, soil microbial species, and diversity index. This study comprehensively analyzes soil microecology from the perspectives of microbial community structure, network analysis, biomarkers, and keystone taxa and provides a theoretical reference for improving the farmland management system of organic tea plantations in Pu'er City, the world's tea source, and similar climate regions.

## 2. Materials and methods

### 2.1. Study area

The study area is located in Pu'er City, Yunnan Province, China. Yunnan Province is a typical region for tea production. The tea plantation area of Yunnan Province accounts for more than 15% of the Chinese tea production area. By 2021, the certified area of organic tea plantations and the certified number of organic products in Yunnan Province were 70,500 ha and 1,406, respectively. The certified area of organic tea plantations and the certified number of organic products in Yunnan Province were the first in China for many consecutive years (Fu et al., 2022). Pu'er is a subtropical region known as the World Tea Source.

The information on the three sampling sites in this study is shown in Table 1. The average altitude of the three sampling sites is 1,326 m. The farmland type is a field with a ladder section (terrace). The soil was classified as *L*a*terite* based on the United States Department of Agriculture's (USDA) textural classification system. The local area has a low-latitude plateau subtropical monsoon climate, with an annual average temperature of 17.8°C and an annual average precipitation of 1,500–1,600 mm. The frost-free period is 325 days, and the annual sunshine hours are 2133.6 h. Before the implementation of organic management (OM), the local soil had the following physical and chemical properties: a pH of 5.13, organic matter content of 24.2 g kg^−1^, total nitrogen of 1.07 g kg^−1^, alkali-hydro nitrogen of 117 mg kg^−1^, available phosphorus of 14.6 mg kg^−1^, available potassium of 64 mg kg^−1^, and slowly available potassium of 321.5mg kg^−1^.

**Table 1:** Introduction of treatments.

**Treatment name**	**Longitude and latitude**	**Management type**	**Planting years**	**OM years**
OM-11	N22°44′, E100°53′	OM	11 years	11 years
CM-11		CM		
OM-16	N22°48′, E100°55′	OM	16 years	14 years
CM-16		CM		
OM-24	N22°45′, E100°52′	OM	24 years	14 years
CM-24		CM		

OM, organic management; CM, conventional management.

### 2.2. Field management and soil sampling

The three tea plantations selected in the study have the same agronomic measures and are relatively close to each other (within 10 km). The distance between the OM and CM in each plantation is ~1 km. In this way, sampling sites were kept under the same climate and soil type, and the differences stemmed from agronomic measures.

The variety of tea trees was *Xueya 100*. The tea plantation was cultivated artificially, and the height of the tea tree was 60–70 cm. The three sampling sites both contained organic management (OM) and conventional management (CM) models (six treatments in total). The management method of OM or CM was consistent between all sampling sites. The organic management methods were as follows: the base fertilization period was from November to December every year, and topdressing was in May. Base fertilizer was 9,000 kg ha^−1^, and topdressing was 3,000 kg ha^−1^. After fertilization, 20–30-cm deep ditches were dug and covered with soil. Organic fertilizer had passed the national organic management standard certification. The organic fertilizer contained 42% organic matter, with a total nutrient content of 6% (N 2.1%, P_2_O_5_ 2.71%, and K_2_O 1.54%) and a pH value of 7.6. The tea plantations used physical methods such as yellow boards and pest control lamps to control pests. The conventional management treatment adopted the production habits of local farmers: the base fertilizer (N-P-K: 25-6-9) was applied in December, and the topdressing (urea) was applied in the middle of May. The base fertilizer was 1,200 kg ha^−1^, and the top dressing was 600 kg ha^−1^. Glyphosate was used for weeding, and acetamiprid was used for pest extermination.

Soil samples were collected in July 2022, and the period was the peak period for tea tree growth. The selected sampling point should avoid the shade of other trees and the side of the road and be close to the middle of the tea plantation to avoid marginal interference. In each treatment, three plots (10 m × 10 m) were selected at the same altitude. The distance between plots should be at least 10 m or more. Five sampling points were set as “plum blossoms” in each plot. The position for soil sampling was selected 10 cm outside the fertilization ditch, and then plant residues were removed on the surface. Bulk soil was collected using a drill with a diameter of 3 cm at a depth of 0–20 cm. On each plot, five soil samples were mixed and homogenized through a 2-mm sieve. In this way, three samples were obtained from every treatment, and a total of 18 samples were obtained from the six tea plantations. Afterward, the soil sample was divided into two parts. One part was stored in a refrigerator at −80°C for the sequencing of microbial metagenomics, and the other part was taken back to the laboratory to measure soil water content (SWC) and for physicochemical analyses.

### 2.3. Measurement index and method

#### 2.3.1. Physicochemical analyses

The SWC was determined using the drying method: weigh 10 g of fresh soil, dry it at 105°C in the aluminum box weighed in advance, and calculate the soil moisture content (Bao, 2000). The organic carbon (SOC) content was determined via a multi-N/C 3100 TOC/TC analyzer (Analytik Jena, Germany). The total nitrogen (TN) content was determined using the Kjeldahl method (Kirk, 1950), and nitrate nitrogen content (N-NO${}_{3}^{-}$) and ammonia nitrogen content (N-NH${}_{4}^{+}$) were determined by Auto Analyzer 3 (Seal company). The content of available phosphorus (AP), total phosphorus (TP), available potassium (AK), and soil pH were measured through standard soil testing procedures (Bao, 2000).

#### 2.3.2. DNA extraction, construction of a PE library, and genome sequencing

According to the E.Z.N.A^®^ Soil DNA Kit (Omega Bio-tek, USA) instructions, soil genomic DNA was extracted. The quality of the DNA extraction was assessed using 1% agarose gel electrophoresis. The concentration and purity of DNA were determined using a micro fluorometer (TBS 380) and a nucleic acid protein meter (NanoDrop ND 2000). DNA fragments (~400 bp in length) were fragmented using an ultrasonic instrument (Covaris M220). The sequencing library was constructed using the NEXTFLEX^TM^ Rapid DNA-Seq Kit (BioScientific USA). Paired-end (PE) sequencing was performed on the Illumina NovaSeq platform (Shanghai Majorbio Technology Co., Ltd.).

#### 2.3.3. Analysis of metagenomics sequencing data

Metagenomics sequencing data used fastp (Version 0.20.0) (Chen et al., 2018) to control the quality of the sequence. Megahit (Version 1.1.2) software (Li et al., 2015) was used to conduct the multiple mixed splicing of the optimized sequence, and more than 300 bp overlap groups (contigs) were screened as the final assembly result. Then, Prodigal (Hyatt et al., 2010) was used to predict the open reading frame (ORF), and genes with nucleic acid lengths of more than 100 bp were screened and translated into amino acid sequences. CD-HIT (Version 4.6.1) software (Fu et al., 2012) was used to cluster the predicted gene sequence. The cluster similarity and coverage of the gene sequence were 90%. The longest gene was taken as the representative sequence of each category to construct a non-redundant gene set. SOAPaligner (Version 2.21) software (Li et al., 2009) was used to compare high-quality reads with non-redundant gene sets and then calculate the gene abundance information with a similarity of over 95% (Lawson et al., 2017).

#### 2.3.4. Statistical analysis and data visualization

Diamond (Version 0.8.35) software (Buchfink et al., 2015, 2021) was used to compare the amino acid sequence of the non-redundant gene set with the NR database. The species annotation results were obtained through the corresponding taxonomic information database, and the species abundance table was constructed at each taxonomic level. According to the results of taxonomic analysis, the structural composition of species or genes in different groups (or samples) would be understood. The relative abundance of bacteria (or fungi) at different classification levels is the ratio of the abundance of each bacteria (or fungi) to the total abundance of bacteria (or fungi).

Sample and microbial species relationship diagrams were visualized using Circos-0.67-7 (http://circos.ca/). The alpha diversity indices (Shannon, Chao, and Heip) based on read numbers at the species level were calculated in QIIME2 (Caporaso et al., 2010). The PCoA analysis based on the read number at the species level used the Binary Jaccard distance algorithm in the R “vegan” package and visualized the results using the “ggplot2” package. The intergroup differences in microorganisms were determined via ANOSIM analysis. ANOSIM analysis was implemented through the R “vegan” package.

This study discovered multi-level biomarkers through linear discriminant analysis effect size (LEfSe). Specifically, the non-parametric Kruskal–Wallis sum-rank test was used to test the species abundance differences between groups, and species with significant differences were obtained. Then, LDA linear discriminant analysis (LDA > 3.0) was used to estimate the degree of impact of these different species on the intergroup differences. The analysis process was performed using LEfSe software (http://huttenhower.sph.harvard.edu/lefse/) (Segata et al., 2011). The correlation coefficient between environmental factors and the alpha diversity index (Spearman) was calculated using the R “psych” package, and the results were calculated using the “ggplot2” package.

The microorganism co-occurrence network was constructed using the Python “Networkx” package. The top 50 species (species level) with abundances were selected in this analysis. Spearman's rank correlation coefficient was adopted in this study. A *p-*value of <0.05 was considered a valid co-occurrence. The correlation threshold was set to 0.5. The visualization of networks and calculation of topology parameters were completed through Gephi 0.9.7. Topology parameters included degree, network diameter, density, modularity, number of communities, average clustering coefficient, average path length, and positive and negative links (Bastian et al., 2009). In this study, the top five species with the highest clustering coefficients were identified as keystone taxa.

Analysis of variance (ANOVA) and least significant difference (LSD) multiple comparison tests (*p* < 0.05) were used for one-way or multivariate analysis of variance. The statistical analysis of the data was conducted in Excel 2016 and SPSS 26 (SPSS Inc., Chicago, USA). The structural equation model was constructed using the partial least squares method. The model construction platform was SmartPLS 3.

## 3. Results

### 3.1. Soil microbial diversity

The community coverage showed that the current sequencing depth covered all the bacteria and fungi in the soil, which can comprehensively reflect the situation of soil bacteria and fungi communities (Table 2). Compared with the CM, the bacterial Chao (richness index) significantly decreased by 5.5% in the OM (*p* < 0.01), while Shannon (diversity index) and Heip (evenness index) had no significant change. The fungal community Chao index reduced (29%) significantly in OM tea plantations, but the Heip and Shannon indices increased (84 and 7%, respectively). The fungi Alpha diversity indexes significantly differed between different management methods (*p* < 0.01).

**Table 2:** Alpha diversity of bacteria and fungi in tea plantation soil.

**Sample**	**Bacteria**				**Fungi**			
	**Shannon**	**Chao**	**Heip**	**Coverage**	**Shannon**	**Chao**	**Heip**	**Coverage**
OM-24	5.63 ± 0.16b	20832 ± 303c	0.013 ± 0.0020b	1	4.14 ± 0.27b	386 ± 2.12d	0.17 ± 0.03a	1
CM-24	5.74 ± 0.41ab	21809 ± 1712bc	0.015 ± 0.0047ab	1	3.81 ± 0.08c	595 ± 9.90c	0.09 ± 0.01b	1
OM-16	6.20 ± 0.08a	23848 ± 446a	0.021 ± 0.0013a	1	4.66 ± 0.09a	662 ± 72.8b	0.15 ± 0.01a	1
CM-16	6.21 ± 0.08a	23868 ± 165a	0.021 ± 0.0017a	1	4.49 ± 0.17a	778 ± 11.3a	0.11 ± 0.02b	1
OM-11	5.44 ± 0.36b	20731 ± 764c	0.012 ± 0.0049b	1	3.61 ± 0.18c	381 ± 29.0d	0.10 ± 0.00b	1
CM-11	5.79 ± 0.16ab	23537 ± 834ab	0.014 ± 0.0020b	1	3.30 ± 0.06d	601 ± 17.7c	0.05 ± 0.00c	1
Locale (L)	^**^	^**^	^**^	—	^**^	^**^	^**^	—
Management (M)	NS	^**^	NS	—	^**^	^**^	^**^	—
L^*^M	NS	^**^	NS	—	NS	^**^	NS	—

OM, organic management; CM, conventional management. ^**^ represents *p* < 0.01. NS represents no significant difference. The same below. Values are means of three replicates ± SD followed by different letters that differ significantly (Turkey's test, *p* < 0.05).

**Figure 1:**
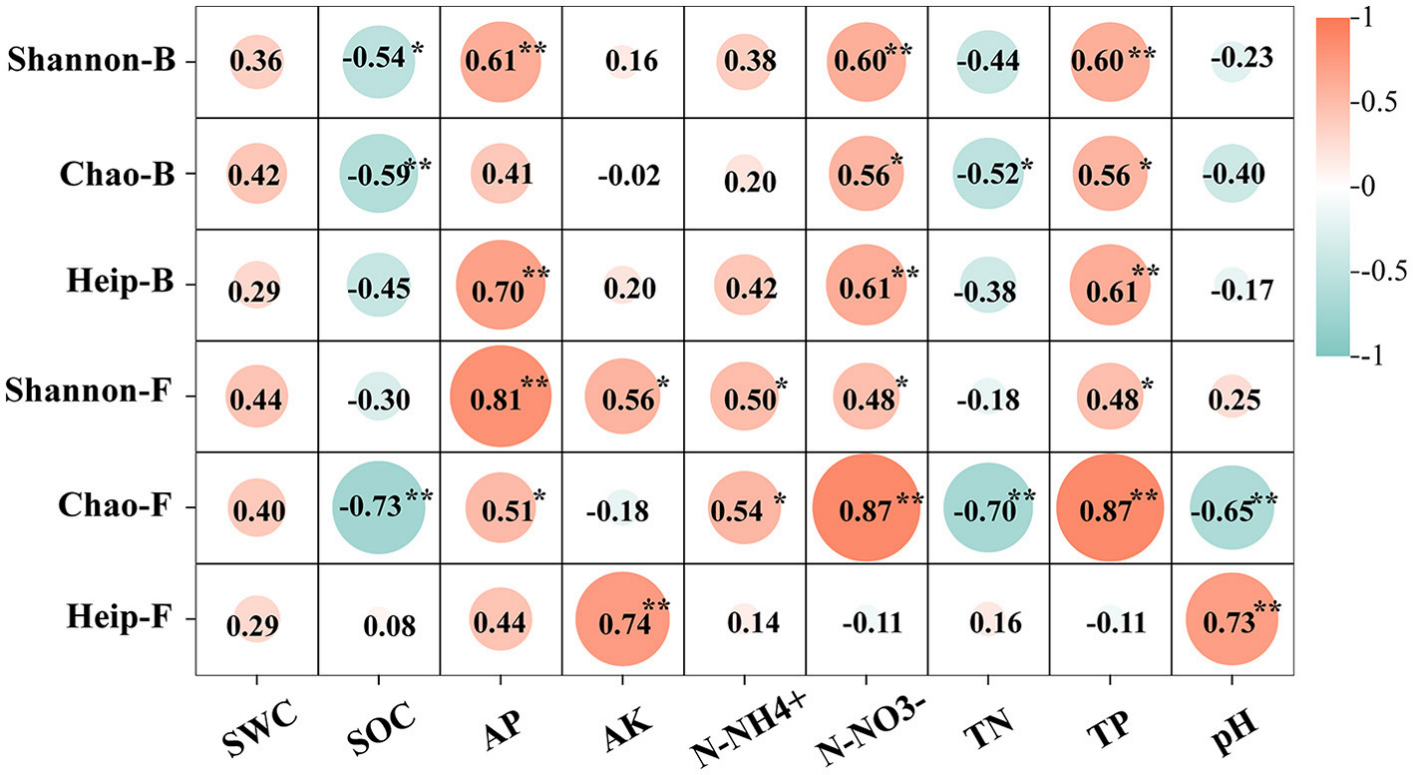
Correlation of environmental factors and bacteria or fungi Alpha diversity index. Shannon-B represents the bacteria's Shannon index. Chao-B represents the bacteria's Chao index. Heip-B represents bacteria's Heip index. Shannon-F represents the Shannon index of fungi. Chao-F represents the Chao index of fungi. Heip-F represents the Heip index of fungi. **Represents 0.001 ≤ *p* < 0.01. *Represents 0.01 ≤ *p* < 0.05. No * representes no relevance. The same below. The values are the correlation coefficient *r*^2^ between the alpha diversity index and environmental factors.

The physical and chemical properties of OM and CM plantation soils are shown in Table 3. The correlation analysis between the alpha diversity index and soil environmental factors showed that most of the environmental factors examined in the study were significantly associated with the alpha diversity index (*p* < 0.05). SOC was significantly negatively correlated with Shannon-B and Chao (Figure 1). There was a significant positive correlation between AP and Shannon, Heip-B, and Chao-F. AK was significantly positively correlated with Shannon-F and Heip-F indices. N-NH${}_{4}^{+}$ influenced Shannon-F and Chao-F positively. TP and N-NO${}_{3}^{-}$ showed a significant positive correlation with the diversity index other than Heip-F. TN influenced Chao negatively. pH was negatively correlated with Chao-F and positively correlated with Heip-F.

**Table 3:** Soil physical and chemical properties of OM and CM.

**Treatment**	**SWC**	**N-NH${}_{4}^{+}$ (mg kg^−1^)**	**N-NO${}_{3}^{-}$(mg kg^−1^)**	**AP (mg kg^−1^)**	**AK (mg kg^−1^)**	**TN (g kg^−1^)**	**TP (g kg^−1^)**	**SOC (g kg^−1^)**	**pH**
OM-11	0.19 ± 0.02	1.97 ± 0.23	3.66 ± 0.37	57.4 ± 2.91	342 ± 11.2	2.55 ± 0.11	0.38 ± 0.04	59.11 ± 0.92	5.46 ± 0.03
CM-11	0.23 ± 0.00	0.71 ± 0.20	5.68 ± 1.39	17.5 ± 2.24	247 ± 8.06	1.66 ± 0.23	0.21 ± 0.01	30.55 ± 1.57	4.51 ± 0.04
ANOVA	^*^	^**^	^*^	^**^	^**^	^**^	^**^	^**^	^**^
OM-16	0.25 ± 0.01	1.83 ± 0.01	9.44 ± 0.06	119 ± 19.9	640 ± 8.69	2.01 ± 0.14	0.43 ± 0.04	33.49 ± 1.95	5.55 ± 0.22
CM-16	0.23 ± 0.01	8.9 ± 1.26	11.2 ± 1.61	106 ± 27.8	232 ± 9.93	1.53 ± 0.08	0.35 ± 0.03	27.18 ± 1.23	4.31 ± 0.07
ANOVA	NS	^**^	NS	NS	^**^	^**^	NS	^**^	^**^
OM-24	0.24 ± 0.00	3.19 ± 0.08	3.10 ± 0.37	41.6 ± 10.0	460 ± 13.9	2.07 ± 0.02	0.32 ± 0.05	36.93 ± 1.34	5.98 ± 0.10
CM-24	0.25 ± 0.01	4.32 ± 0.33	10.32 ± 0.78	34.5 ± 3.92	315 ± 9.46	2.08 ± 0.39	0.31 ± 0.01	28.39 ± 1.90	4.19 ± 0.01
ANOVA	NS	^**^	^**^	NS	^**^	NS	NS	^**^	^**^

SWC, soil water content; N-NH${}_{4}^{+}$, ammonia nitrogen content; N-NO${}_{3}^{-}$, nitrate nitrogen content; AP, available phosphorus content; AK, available potassium content; TN, total nitrogen content; TP, total phosphorus content. Values are means of three replicates ± SD. ^**^ represents *p* < 0.01. ^*^ represents 0.01 ≤ *p* < 0.05. NS represents no significant difference.

**Figure 2:**
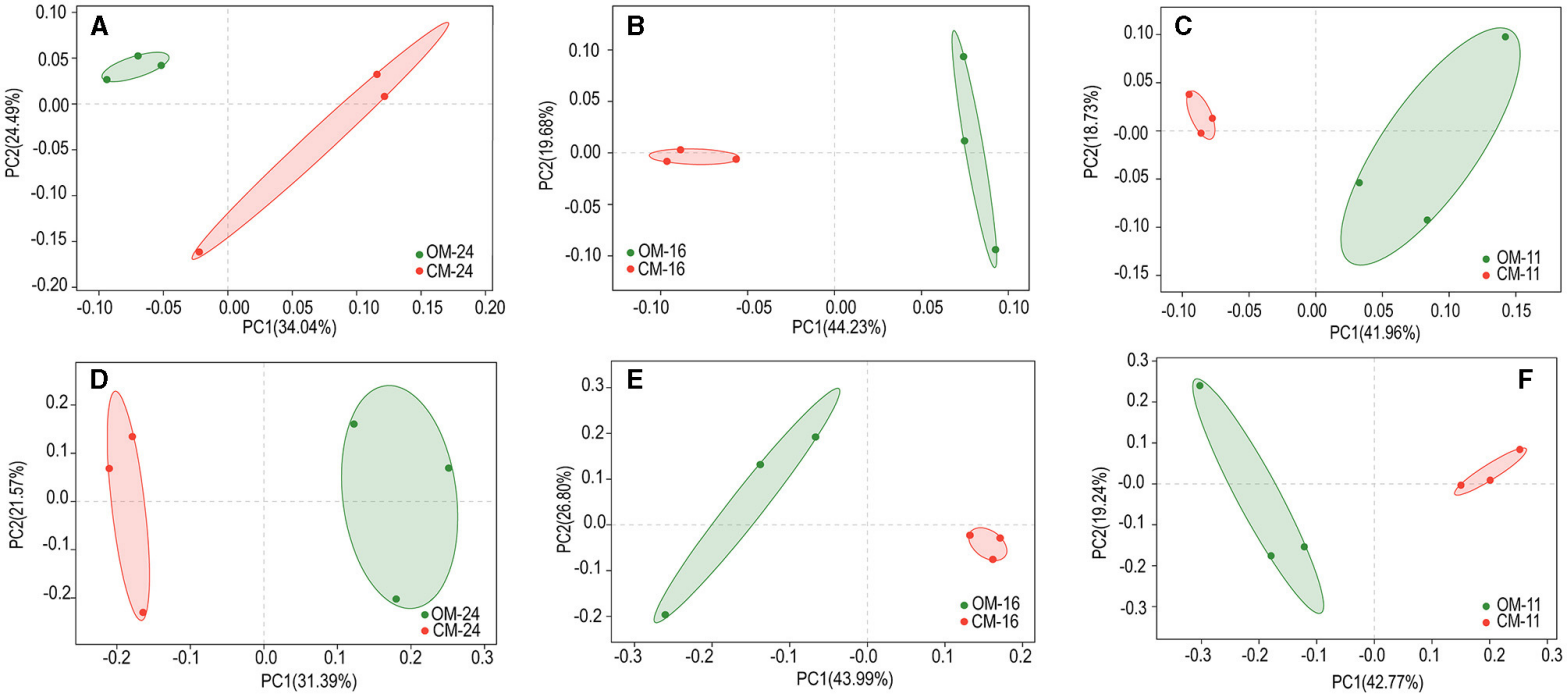
Bacterial **(A–C)** and fungal **(D–F)** Beta diversity of tea plantations soil in different management modes. OM, organic management; CM, conventional management.

The study was based on PCoA analysis (Binary-Jaccard) at the species level to compare the differences in the structure of bacterial or fungal communities under two management methods (Figure 2). The first and second coordinate axes represented the contribution values of the first and second principal components to the treatment difference. In the 24-year plantation, the first and second principal components accounted for 58.53 and 52.96% of the variance in bacterial and fungal communities, respectively. In the 16-year plantation, these figures were 63.91 and 70.79%, while in the 11-year plantation, they were 60.69 and 62.01%. Bacterial and fungal communities were clearly separated between organic and conventional management.

### 3.2. Bacterial community structure

**Figure 3:**
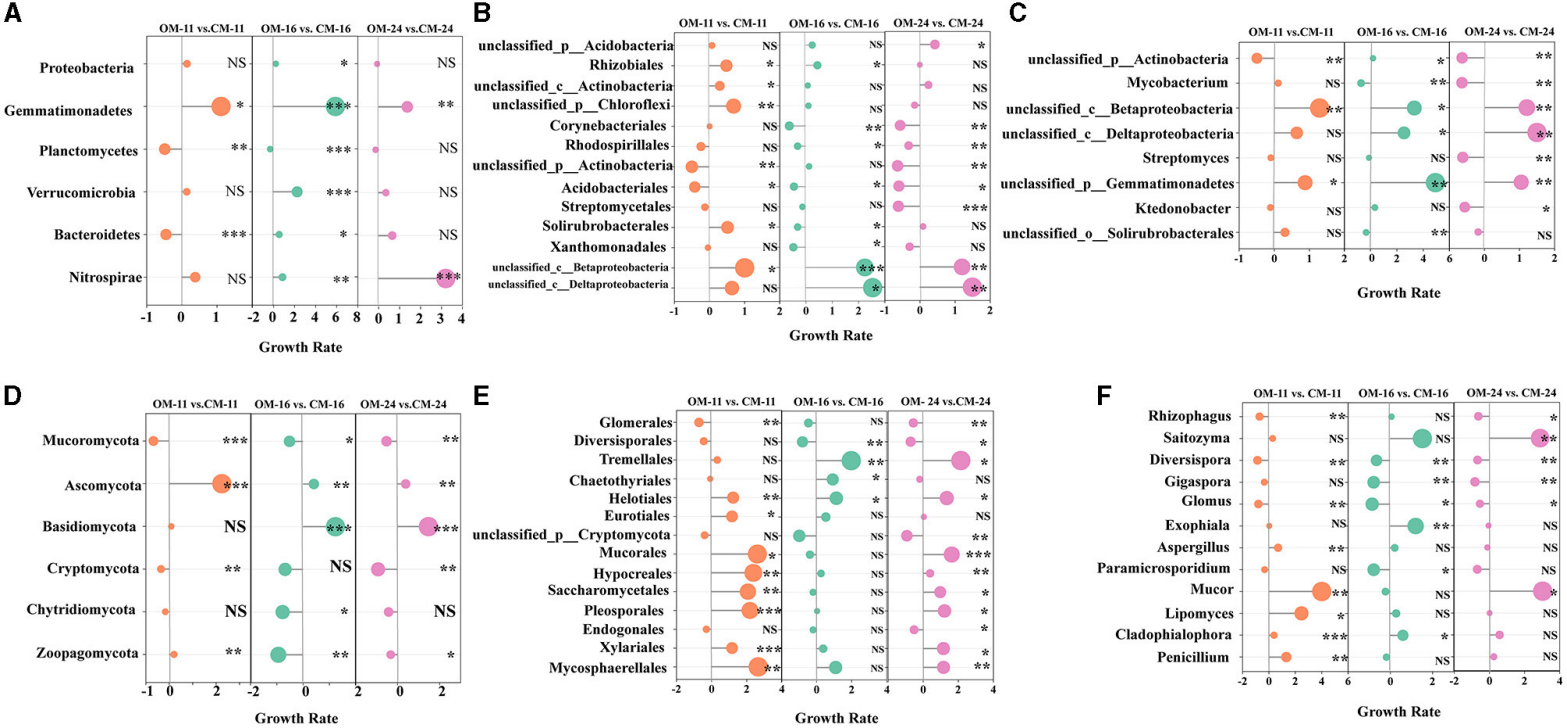
The growth rate of species relative abundance of OM relative to CM. **(A)** Bacterial phylum level **(B)** Bacterial order level **(C)** Bacterial genus level **(D)** Fungal phylum level **(E)** Fungal order level **(F)** Fungal genus level. The size of points represents the numerical value. NS represents no significant difference. ***Represents *p* < 0.001. **Represents 0.001 ≤ *p* < 0.01. *Represents 0.01 ≤ *p* < 0.05. OM, organic management; CM, conventional management.

In this study, the non-redundant gene set annotated by the NR database was used to compare the relative abundance of bacteria species in tea plantation soil under different management modes. An analysis of variance between OM and CM was performed on bacterial phyla with a relative abundance of >1%. Among the dominant bacterial phyla (relative abundance ≥ 10%) (Supplementary Figure S1A), compared with CM-16, the relative abundance of *Proteobacteria* in OM-16 significantly increased by 24.3% (*p* < 0.05) (Figure 3A). In the non-dominant bacterial phylum (relative abundance < 10%), *Gemmanimonadetes* showed 1.1 times (*p* < 0.05), 5.89 times (*p* < 0.001), and 1.37 times (*p* < 0.01) higher levels in OM-11, OM-16, and OM-24 compared to the CM treatment, respectively. *Planctomycotes* significantly decreased by 29% (*p* < 0.001) and 48% (*p* < 0.01) under OM-16 and OM-11, respectively. *Verrucomimicrobia* significantly increased by 2.26 times under OM-16 (*p* < 0.001). *Bacteroidetes* significantly increased by 56% (*p* < 0.05) in OM-16 compared to CM-16 but decreased by 44% (*p* < 0.001) in OM-11 compared to CM-11. OM could improve the relative abundance of the *Nitrospirae* community, especially when the OM-24 treatment was three times higher than that of CM-24.

The dominant bacterial order (relative abundance ≥ 10%) included *Rhizobiale*s and the unclassified bacterial orders of *Acidobacteria, Chloroflexi*, and *Actinobacteria* (Supplementary Figure S1C). An analysis of variance between OM and CM of bacteria orders with relative abundance in the top 15 was conducted between OM and CM. The relative abundance of unclassified bacterial orders of *Acidobacteria* was significantly higher in OM-24 than in CM-24 (43%, *p* < 0.05) (Figure 3B). The relative abundance of the unclassified bacterial orders of *Chloroflexi, Actinobacteria, and Rhizobiales* in OM-11 was significantly higher than that in CM-11. The growth rates were respectively 69% (*p* < 0.05), 30% (*p* < 0.05), and 49% (*p* < 0.05). The non-dominant bacteria orders (relative abundance < 10%) in the three tea plantation locations were the same, mainly including *Corynebacteriales, Rhodospirillales, Acidobacteriales, Ktedonobacteria*, and so on. The *Acidobacteriale* levels of the OM were significantly lower than those of the CM. Compared with CM-24 and CM-16, OM-24 and OM-16's *Corynebacteriales* and *Rhodospirillales* were significantly lower.

The dominant bacteria genus (relative abundance ≥ 10%) in the three sampling sites were unclassified bacteria genera under *Acidobacterium, Actinobacia*, and *Chloroflex* (Supplementary Figure S1E). An analysis of the variance of bacteria genera with relative abundance in the top 15 between OM and CM was conducted. The dominant bacterial genus had no significant difference between the OM and CM (Figure 3C). The non-dominant bacteria genus (relative abundance < 10%) included unclassified bacteria genera of *Acidobacterium, Chloroflexi, Actinobacia, Alphaproteobacteria, Betaproteobacteria, Deltaproteobacteria, Streptomyces, Bradyrhizobium, Mycobacterium*, and so on (Supplementary Figure S1E). Organic management changed the relative abundance of non-dominant bacteria in tea plantations. As shown in Figure 3C, the relative abundance of *unclassified_c__Betaproteobacteria* significantly increased by 1.3 times (*p* < 0.01), 3.3 times (*p* < 0.05), and 1.2 times (*p* < 0.01) in OM-11, OM-16, and OM-24, respectively. The *unclassified_c__Deltaproteobacteria* in OM-16 and OM-24 also showed an increasing trend, with 2.6 times (*p* < 0.05) and 1.5 times (*p* < 0.01) compared to CM, respectively. Compared with the CM, *Mycobacterium* significantly decreased by 70 and 63% (*p* < 0.05) in OM-16 and OM-24, respectively. *Streptomyces* significantly decreased by 61% (*p* < 0.01) in OM-24 (Figure 3C).

### 3.3. Fungal community structure

At the dominant phylum level (relative abundance ≥ 10%) (Supplementary Figure S1B), analysis of variance between OM and CM was performed on fungal phyla with a relative abundance of >1%. In the OM, the relative abundance of *Mucoromycota* significantly decreased by 56% (*p* < 0.05) and that of *Ascomycota* significantly increased by a fold (*p* < 0.01) (Figure 3D). The relative abundance of *Basidiomycota* in OM-24 and OM-16 significantly increased by 1.48 and 1.23 (*p* < 0.001), respectively, compared to the CM. Among non-dominant fungal phyla (relative abundance < 10%), *Cryptomycota, Chytidiomycota*, and Z*oopagomycota* showed a certain degree of decrease in OM. At the fungi orders, *Glomerales, Diversisporales*, and *Tremella* (relative abundance ≥ 10%) were the dominant orders (Supplementary Figure S1D). An analysis of variance between OM and CM of fungi orders with relative abundance in the top 15 was conducted. Compared to CM, the relative abundance of *Glomerales* and *Diversesporales* in OM decreased, while the relative abundance of *Tremella* increased (Figure 3E). Non-dominant orders (relative abundance < 10%) included *Chaetothyriales, Helotiales, Eurotiales, unclassified_p__Cryptomycota, Mucorales, Hypocreales, Saccharomycetales, Pleosporales*, and so on. The relative abundance of *Chaetothyriales* in OM-16 significantly increased by 93% (*p* < 0.05), while the relative abundance of *Helotiales* doubled (*p* < 0.05). The relative abundance of *Mucorales, Hypocreales, Saccharomycetales, and Pleosporales* significantly increased in OM-11 and OM-24 (*p* < 0.05).

At the genus level of fungi, the dominant genera (relative abundance ≥ 10%) include *Rhizophagus, Saitozyma*, and *Diversispora* (Supplementary Figure S1F). An analysis of variance between OM and CM of fungi genera with relative abundance in the top 15 was conducted. Compared with CM, the relative abundance of *Rhizophagus* in OM-11 and OM-24 decreased significantly by 70% (*p* < 0.01) and 62% (*p* < 0.05) (Figure 3F). Diversispora of OM-11, OM-16, OM-24 significantly reduced by 86% (*p* < 0.001), 65% and 66% (*p* < 0.01), respectively. The relative abundance of *Saitozyma* significantly increased by 3-fold in OM-24 (*p* < 0.01). The non-dominant genera (relative abundance < 10%) mainly included *Gigaspora, Glomus, Exophiala, Aspergillus, Paramicrosporidium, Mucor, Lipomyces*, and so on. The relative abundance of *Gigaspora, Glomus*, and *Paramicrosporium* significantly decreased (*p* < 0.05) or did not show significant changes in OM. However, the relative abundance of *Exophiala, Aspergillus, Mucor, and Lipomyces* significantly increased (*p* < 0.05) or remained unchanged in OM.

### 3.4. The biomarkers of the organic tea plantation

**Figure 4:**
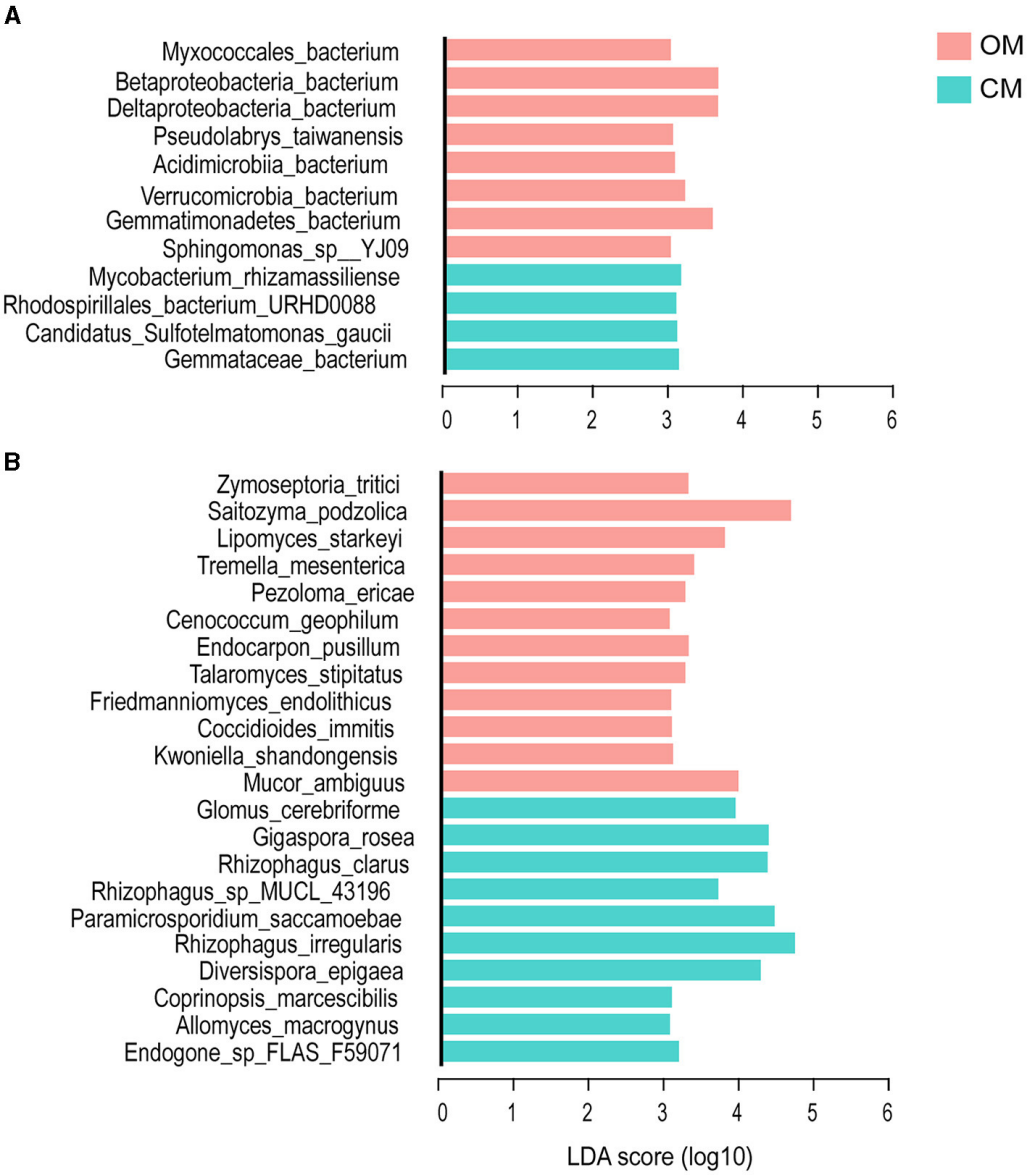
The biomarkers of the tea plantation soil under OM and CM. **(A)** Bacteria and **(B)** fungi. OM, organic management; CM, conventional management.

In this study, LEfSe was used to further clarify the soil biomarker groups under different management modes in order to evaluate the species causing the difference between OM and CM by setting a linear discriminant analysis (LDA) value. When the LDA score was >3.0, there were significant differences between OM and CM in 12 bacterial species and 22 fungal species (*p* < 0.05) (Figure 4). In this study, these different species were considered the biomarker group. “OM” contained eight bacteria and 12 fungal biomarkers, while CM contained four bacteria and 10 fungal biomarkers.

Among the bacteria biomarker group in OM, *Betaproteobacteria_ bacterium* was the species with the highest LDA score (LDA score = 3.655, *Proteobacteria*) (Figure 4A). More than 50% of the members of the bacteria biomarker group belonged to the *Proteobacteria*. Other members came from the *Germatimonadetes, Verrucomicrobia*, and *Actinobacteria*. The four members of the biomarker group of CM were derived from *Actinobacteria, Proteobacteria, Planctomycotes*, and *Acidobacteria. Mycobacterium_ rhizamasilianse* (*Actinobacteria*) had the highest LDA score (3.159).

*Saitozyma_ Podzolica* had the highest LDA score among the soil fungi biomarker group in OM (LDA score = 4.608, *Basidiomycota*). Other biomarkers were derived from *Ascomycota and Mucormycota* (Figure 4B). Most (67%) of the biomarkers belonged to *Ascomycota*. The highest LDA score for the biomarker group of soil fungi in CM was *Rhizophagus_ Iregularis* (LDA score = 4.66, *Mucormycota*), and the rest of the biomarkers were derived from *Cryptomycota* and *Basidiomycota*. A total of 70% of the biomarkers of soil fungi in CM belonged to *Mucormycota*.

### 3.5. Taxonomic co-occurrence networks

**Figure 5:**
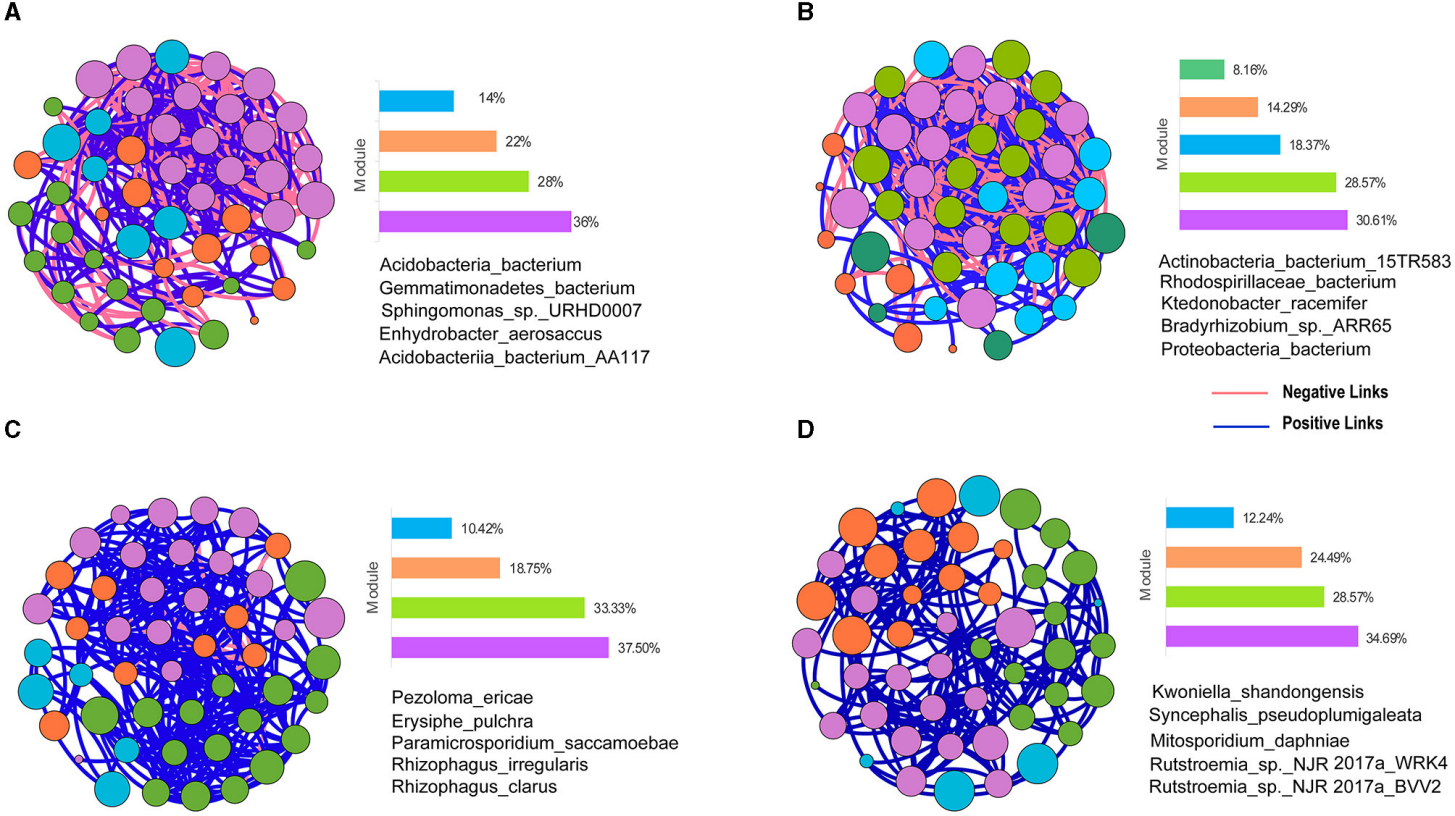
Taxonomic co-occurrence networks of bacterial and fungal species in the OM and CM. **(A)** Bacterial taxonomic co-occurrence network in the OM. **(B)** Bacterial taxonomic co-occurrence network in the CM. **(C)** Fungal taxonomic co-occurrence network in the OM. **(D)** Fungal taxonomic co-occurrence network in the CM. OM, organic management; CM, conventional management. Nodes in the figure represent species. The size of nodes represents the size of the clustering coefficient. Nodes of the same color form a module. The bar chart represents the proportion of each module in the whole symbiotic network. The red lines between nodes indicate a negative correlation between the two species, and the blue lines indicate a positive correlation. The species shown in the figure are keystone taxa in the co-occurrence network.

To explore the health condition of soil bacterial and fungal ecosystems under the OM and CM, the study used taxonomic co-occurrence networks to analyze the symbiotic patterns of bacteria and fungi in the top 50 soil species abundances (Figure 5).

From the perspective of bacterial network topology parameters, compared to the CM, OM reduced the total number of connections (edges) and the connectance (degree) of bacteria's co-occurrence network (Table 4). The average degree of bacterial co-occurrence networks in the CM was 17.4, which was 1.3 times higher than the OM, indicating that the bacterial co-occurrence networks in the CM were more complex than the CM. The diameter and density of the soil bacterial co-occurrence network were higher in CM than in OM. Although the number of nodes in the bacterial network was the same between the OM and CM, there were more diverse connections between nodes in the CM.

**Table 4:** Topological network parameters.

**Features**	**OM**		**CM**	
	**Bacteria**	**Fungus**	**Bacteria**	**Fungus**
Nodes	50	48	49	49
Edges	330	278	426	194
Degree	13.2	11.6	17.4	8.0
Network diameter	5	4	7	7
Density	0.269	0.246	0.362	0.165
Modularity	0.259	0.339	0.151	0.552
Number of communities	4	4	5	4
Average clustering coefficient	0.607	0.597	0.737	0.622
Average path length	2.08	2.067	2.182	2.737
Positive links	64.24%	96.04%	53.76%	100%
Negative links	35.76%	3.96%	46.24%	0.00%

OM, organic management; CM, conventional management.

From the perspective of fungal network topology parameters, compared to the CM, the OM increased the total number of connections (edges) and the connectance (degree) (Table 4). The average degree of fungal co-occurrence network in the OM was 11.59, which was 1.5 times higher than that in the CM. It indicated that the fungal networks were more complex in the OM. The diameter of the soil fungal co-occurrence network under the CM was higher than that under the OM, but the number of edges under the OM was higher than that under the CM, which led to a higher density of the OM's fungal network structure.

The modularity of the bacterial network in OM was higher than that in CM, while the opposite was true for fungi. The average clustering coefficient of the soil bacterial and fungal co-occurrence network nodes in CM was higher than that in OM, indicating that the species in the co-occurrence network of CM had closer connections with their neighboring species compared to those in OM (Table 4). The edge number of the bacterial co-occurrence network in CM was higher than that in OM. However, the proportion of positive correlation between bacterial species in OM was 11% higher than that in CM, indicating that OM was more conducive to the establishment of a positive correlation between bacterial species. The edge number of the fungal co-occurrence network in OM was higher than that in CM, and the relationships between fungal species in CM were all positive. There was also a nearly 4% negative correlation between OM species. It indicated that OM led to a certain degree of negative correlation between fungal community species.

The top five species with multiple correlations with other microorganisms were classified as keystone taxa based on the clustering coefficient. The contribution of these keystone taxa to the community network structure was due to their influence, not abundance (Figure 5, Supplementary Table S1).

### 3.6. Correlation between management mode, environmental factors, and soil microorganisms

**Figure 6:**
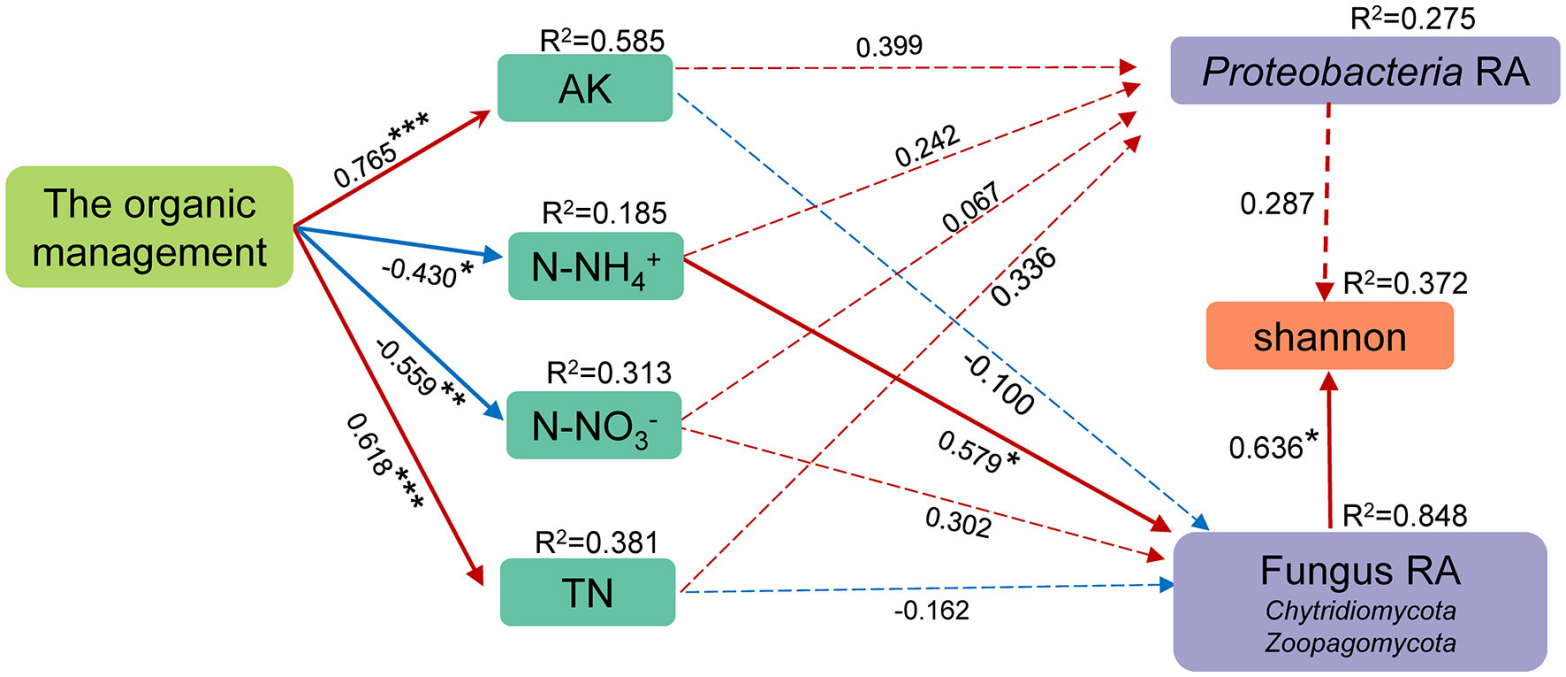
The structural equation model (SEM) for the management mode, soil environmental factors, the relative abundance of bacteria and fungi at the phylum level, and the Shannon index. The value above the SEM line represents the path coefficient. RA represents relative abundance. The red line represents the positive path coefficient, and the blue line represents the negative path coefficient. ***Represents *p* < 0.001. **Represents 0.001 ≤ *p* < 0.01. *Represents 0.01 ≤ *p* < 0.05. The solid line represents a significant correlation between two factors, while the dashed line represents a non-significant correlation.

The relationship between management mode, environmental factors, and soil microorganisms was further analyzed by constructing a PLS-SEM model (Figure 6). First, the autocorrelative environmental factors were removed (VIF > 10), which preserved the less interacting SWC, AK, AP, N-NH${}_{4}^{+}$, N-NO${}_{3}^{-}$, and TN. Then, the above environmental factors, phylum-level species with significant differences in relative abundance between OM and CM, and the Shannon index were included in PLS-SEM. The indicators with weights of <0.2 and VIF > 10 were removed from the PLS-SEM model. SWC AP had no correlation with the management mode, so they were removed. The results of PLS-SEM showed that all latent variables had discriminant validity. The organic management had a positive correlation with AK and TN and a significant negative correlation with N-NO${}_{3}^{-}$ and N-NH${}_{4}^{+}$. However, only N-NH${}_{4}^{+}$ provided a significant impact on soil microorganisms, that is, N-NH${}_{4}^{+}$ affected the relative abundance of *Chytidiomycota* and *Zoopagomycota* in the soil and then further positively affected the Shannon index of soil microorganisms.

## 4. Discussion

Microorganisms are omnipresent and abundant, and their genetic and metabolic diversities are the main participants in the biogeochemistry process (Stott and Taylor, 2016; Essel et al., 2019; Ney et al., 2020). Additionally, their body size is small, and they respond quickly to environmental changes. Based on these characteristics, microbes can be used as excellent biological indicators to characterize environmental disturbance and soil quality (Karimi et al., 2017). The present study used metagenomics sequencing technology to evaluate the microbial community composition and co-occurrence network of three tea plantations under organic and conventional management to understand the soil quality of tea plantations from the perspective of soil microorganisms. In this study, there were significant differences in soil microorganisms in tea plantations between organic and conventional management.

### 4.1. Organic management induced positive changes in microbial alpha diversity in tea plantation soil

The alpha diversity of microorganisms can quantify the diversity characteristic of microorganisms in the soil, which plays a key role in assessing soil quality and the sustainable development of farmland (Lemanceau et al., 2015; Delgado-Baquerizo et al., 2016). There was controversy in previous studies regarding alpha diversity. Previous studies have shown that organic management increased the diversity of soil microorganisms (Kundel et al., 2020; Suyal et al., 2021), remained unchanged (Sugiyama et al., 2010), or even decreased it (Bonanomi et al., 2016; Karanja et al., 2020). This study indicated that OM improved the Shannon index of fungi, while there was no significant change in bacteria. The richness (Chao) of bacteria and fungi was reduced in the OM compared to the CM (Table 2). The result was similar to most research (Bonanomi et al., 2016; Kundel et al., 2020; Suyal et al., 2021). However, some scholars believed that community composition was more important than diversity in soil ecological function due to functional equivalence or redundancy between microorganisms. Therefore, the diversity evaluation of microbial communities needs to be combined with community composition (Bonanomi et al., 2016).

The Heip index reflects the evenness of soil microbial communities. The higher the Heip index, the more uniform the distribution of microbial communities and the more stable soil microecology is. The higher evenness of microbial communities in organic management indirectly characterizes the high stability of soil microecology (Sugiyama et al., 2010). The stability of soil microecology is an important indicator of soil quality (Seybold et al., 1999). The present results were similar to them. The organic management improved the fungus evenness in the soil of tea plantations (Table 2). The lower evenness of soil microorganisms in CM may be due to the increased interference of soil microorganisms caused by the application of insecticides, fertilizers, and herbicides (Sugiyama et al., 2010).

### 4.2. Organic management increased the abundance of beneficial microorganisms in tea plantation soil

The composition of soil microbial communities and the role of species in soil are of great significance for soil quality evaluation (Sugiyama et al., 2010). *Gemmatimonadetes* can degrade soil pollutants and organic matter, producing alkaline phosphatase in the soil (Ding et al., 2016; Durrer et al., 2021). *Gemmatimonadetes* is a eutrophic bacteria that needs enough nutrients (Ghosh et al., 2016). Therefore, the increase in the relative abundance of *Gemmatimonadetes* was regarded as a sign of sufficient nutrients. In this research, *Gemmatimonadetes* in OM was significantly increased, which indirectly suggested that organic management might increase soil nutrient content in tea plantations (Figure 3). To verify this hypothesis, soil nutrients were tested and showed that organic management promoted an increase in soil total nitrogen (TN), organic carbon (SOC), and available potassium content (AK) (Table 3).

*Proteobacteria* was the dominant phylum in organic tea plantations (with a relative abundance of over 30%), with a higher relative abundance compared to conventional management (Figure 3; Supplementary Figure S1A). Biomarkers are often used in microbial research to identify species that cause significant differences between groups. These biomarkers are evaluated based on species abundance, using LEfSe as the method of analysis. In the study, more than half of the bacterial biomarkers originated from the *Proteobacteria* (Figure 4), indicating that the species in the *Proteobacteria* were key species that caused soil microecology differences between OM and CM. *Proteobacteria* mainly include *Alphaproteobacteria, Betaproteobacteria, Deltaproteobacteria*, and *Gammaproteobacteria* (Dedysh et al., 2004; Ghosh et al., 2016). *Pseudolabs_ Taiwanensis* was a bacterial biomarker belonging to *Rhizobiales (Alphaproteobacteria). Rhizobiales* have biological control and nitrogen fixation functions, often coexisting with leguminous plants, and can persist in soil (Bastida et al., 2016; Degrune et al., 2017).

In addition, in this study, the relative abundance of *Rhizobiales* in OM tea plantations was significantly increased compared to CM, possibly due to the leguminous weeds included in organic tea plantations that promote the symbiosis of nitrogen-fixing bacteria. *Rhodospirillales* belonging to *AlphaProteobacteria*, including *Azospirillum* and *Rhodospirillum*, can promote plant growth through nitrogen fixation and hormone synthesis (Souza et al., 2013). Our results suggested the relative abundance of *Rhodospirillales* in OM was significantly reduced (Figure 3). *Betaproteobacteria_Bacterium* and *Deltaproteobacteria_Bacterium* are bacterial biomarkers that belong to *Betaproteobacteria* and *Deltaproteobacteria*, respectively. Aerobic ammonia oxidation mediated by *Betaproteobacteria* is a rate-limiting step in the nitrification process related to plant nitrogen availability, nitrate leaching into groundwater, or N_2_O emissions (Di and Cameron, 2016). *Deltaproteobacteria* promote nitrogen and organic matter cycling in soil (Zhao et al., 2019). In addition, this research also indicated that the relative abundance of unclassified genera in *Betaproteobacteria* and *Deltaproteobacteria* significantly increased in the OM compared to the CM (Figure 3). *Nitrospirae* is an aerobic bacterium with high nitrification activity. It is an important bacterium involved in the oxidation of nitrite in the soil nitrogen cycle (Daims et al., 2015; Durrer et al., 2021). Our result suggested that the relative abundance of *Nitrospirae* was significantly increased, especially in the OM-24, which increased by three times compared to conventional management (Figure 3). The result is that the longer the organic management period, the higher the nitrogen availability, which induces an increase in *Nitrospirae* (Wang et al., 2020).

In summary, most of the biomarkers in OM were related to soil nitrogen metabolism. It indicated that there may be significant differences in soil nitrogen metabolism between OM and CM and that nitrogen metabolism performs better in organic tea plantations. The results of PLS-SEM can also precisely explain this phenomenon. OM reduced N-NH${}_{4}^{+}$ in the soil, increasing the relative abundance of *Proteobacteria* (Figure 6).

*Streptomyces* can produce antibiotics, plant growth hormones, and extracellular enzymes, enhancing the plant's resistance to biotic and abiotic stresses (Ponmurugan and Saravanan, 2013; Zhu et al., 2019). Previous studies have shown that *Streptomyces* preparations can maintain a good level of yield and quality in tea plants infected with bird's eye spot or red rust disease (Gnanamangai and Ponmurugan, 2012; Ponmurugan and Saravanan, 2013). Currently, the relative abundance of *Streptomyces* in the OM-24 is lower than that in the CM-24 (Figure 3). It was due to the acidophily of *Streptomyces*, which was an important factor in its better biological control effect in tea plantations (Gnanamangai and Ponmurugan, 2012). Soil acidification is a universal problem in perennial tea plantations (Rothenberg et al., 2022). In our study, the OM alleviated the problem of soil acidification in conventional tea plantations (Table 3), which explained the significant decrease in the abundance of *Streptomyces* in organically managed tea plantations.

*Ascomycota* and *Basidiomycota* are mainly saprophytic nutrition types. They are related to the degradation of crop residues, and play an important role in soil carbon, nitrogen cycle, and other nutrient cycles (Bastida et al., 2016; Li et al., 2017; Zheng et al., 2018). In the present study, fungi biomarkers in the OM come from *Ascomycota*. The relative abundance of *Ascomycota* and *Basidiomycota* increased significantly in the OM (*p* < *0.05*), which was similar to the previous studies (Ding et al., 2017). It was the large input of organic matter, such as weeds and crop residues, in organic tea plantations that accelerated the reproduction of *Ascomycota* and *Basidiomycota*.

*Eurotiales, Mucorales, Hypocreales, Saccharomycetes*, and *Tremelales* are free-living saprotrophic fungi (Vargas-Gastelum et al., 2015). The majority of species in the *Eurotiales* and *Hypoceras* can produce N_2_O (Mothapo et al., 2015). In our study, *Hypoceras* and *Eurotiales* significantly increased in OM, compared to CM. Further research is needed on their contribution to N_2_O emissions. *Rhizophagus* is one of the AMFs, consuming less carbon but helping host plants absorb more phosphorus (Stefani et al., 2020). Our results suggested that *Rhizophagus* was found as a dominant fungal genus in OM tea plantations, and its relative abundance was significantly lower than that in CM tea plantations. This result may be because less organic carbon in conventional soil management attracts more low-carbon-consuming species. In summary, organic management increased the soil fungi that degrade plant residues and turnover nutrients in tea plantations.

### 4.3. Organic management promoted the stability of soil microecology in tea plantations

Microorganisms do not exist in isolation in soil ecosystems. A microbial species often interacts with other species in multiple ways. These interactions can occur simultaneously and in space, forming a network of interactions (Karimi et al., 2017). Microbial symbiotic networks provide an overall perspective for ecosystems because they integrate the direct and indirect effects of environmental changes on species diversity, taxonomy composition, and relationships between species within communities. A growing number of studies have taken microbial symbiotic networks as indicators of soil quality (Berry and Widder, 2014; Wang et al., 2020; Guseva et al., 2022; Xue et al., 2022; Yang et al., 2023).

The co-occurrence network was constructed by calculating the correlation coefficients between species. When species were significantly correlated, two species were connected by a line. The edge number of bacterial co-occurrence networks was reduced in the CM compared to the OM, but the positive correlation ratio between bacterial species in the OM was 11% higher than that in the CM. It indicated that the OM was more conducive to the establishment of collaborative relationships between bacterial species (Table 4). Modules are considered ecological niches or functional units that constitute different ecological processes in a community (Zhang et al., 2018). In this study, organic management led to a higher modularity of co-occurrence networks (Table 4). The increase in the modularity of microbial networks can indicate that microbial communities have formed more refined ecological niches and functional units (Wang L. X. et al., 2021). The lower modularity of bacterial co-occurrence networks in the CM showed that microbial functional diversity was inhibited, which may be due to the application of pesticides and fertilizers in the CM. Compared with the CM, the fungal co-occurrence networks in the OM had a higher density, which indicated that the OM increased the diversity of species relationships. The OM has increased the number of connections (edges) and the degree of connectivity between fungal network nodes. It proved that the OM increased the complexity of fungal co-occurrence networks and promoted the exchange of metabolites and information between species (Table 4). The result was consistent with that of the study by Chen et al. (2022). In addition, there was also a partial negative correlation between fungal species in the OM, which was not found in the CM (Table 4). This negative correlation means that there is competition, exclusion, or adaptation to ecological niches among species (Cuartero et al., 2022). However, these negative correlations promote the stability of the microbial network of the soil ecosystem because they compensate for the overexpression of some members. Overexpression may lead to the instability of the network (Coyte et al., 2015). Therefore, organic management can protect soil microbial functional diversity and promote the stability of soil ecological networks compared to conventional management.

In the co-occurrence network of soil microorganisms, the clustering coefficient represents the connection between a node and its neighboring nodes. The larger the clustering coefficient, the more important the node is. The more important nodes are regarded as keystone taxa. The top five species ranked by clustering coefficient in our study are called keystone taxa (Figure 5). Banerjee et al. (2018) defined keystone taxa as “highly connected taxa that individually or in a guild exert considerable influence on microbiome structure and functioning irrespective of their abundance across space and time.” All the bacterial keystone taxa in the organic tea plantations belong to *Acidobacteria, Gemmatimonadetes, and Alphaproteobacteria*. According to previous research, *Acidobacteria* participate in the nitrogen cycle and promote the conversion of nitrate and nitrite (Liu et al., 2017). *Alphaproteobacteria* are related to the nitrogen fixation process in soil (Basit et al., 2014).

The fungal keystone taxa in the OM included three types of mycorrhizal fungi (*Pezoloma_ericae, Rhizophagus irregularis*, and *Rhizophagus_clarus*). To our knowledge, mycorrhizal fungi play an important role in improving soil quality, nutrient cycling, and alleviating stress (Fei et al., 2022). *Erysiphe_ Pulchra* originates from *Erysiphe. Erysiphe* is a pathogen of powdery mildew, infecting tea trees (*Litsea Coreana Levl.Var.Lanuginose*) (Zhu et al., 2007). *Paramicrosporidium saccamoebae* was described as an intranuclear parasite of *Saccamoeba* (Quandt et al., 2017). It is necessary to further study their pathogenicity through field observation and pathogenicity verification in the future. In summary, we inferred that organic management may promote soil nitrogen metabolism and nutrient uptake by tea plants. This conjecture still needs our later research to verify it.

## 5. Conclusions

Organic management increased the relative abundance of microorganisms related to nutrient metabolism and plant residue degradation and improved the stability of the microbial ecological network in tea plantation soil. Organic management induced positive changes in microbial alpha diversity in tea plantation soil. Organic management impacted N-NH${}_{4}^{+}$ negatively, which increased the relative abundance of fungi, thereby positively affecting the Shannon index. Overall, organic management can increase beneficial microorganisms and promote the stability of microecological networks. The study can provide a theoretical reference for the implementation of organic planting systems in Yunnan Province, China. The existence of pathogens and parasites also reminds us that there is a need to further improve the supporting agricultural management measures for organic tea plantations in the future.

## Data availability statement

The original contributions presented in the study are included in the article/Supplementary material, further inquiries can be directed to the corresponding authors.

## Author contributions

XH, WG, YC, JC, and PS: conceptualization. XH, YZ, and PL: methodology and investigation. XH: formal analysis, visualization, and writing original draft preparation. WG, YC, and JC: writing review and editing. WG and YC: funding acquisition and resources. WG, YC, JC, and PS: supervision. All authors contributed to the article and approved the submitted version.
