# Supplementary material for: Organic management increases beneficial microorganisms and promotes the stability of microecological networks in tea plantation soil
Source: Front Microbiol. 2023 Sep 19;14:1237842. doi: 10.3389/fmicb.2023.1237842 (PMC10546928; doi:10.3389/fmicb.2023.1237842)
Supplement: Supplementary file 1 [file Data_Sheet_1.docx]

**Supplemental file**


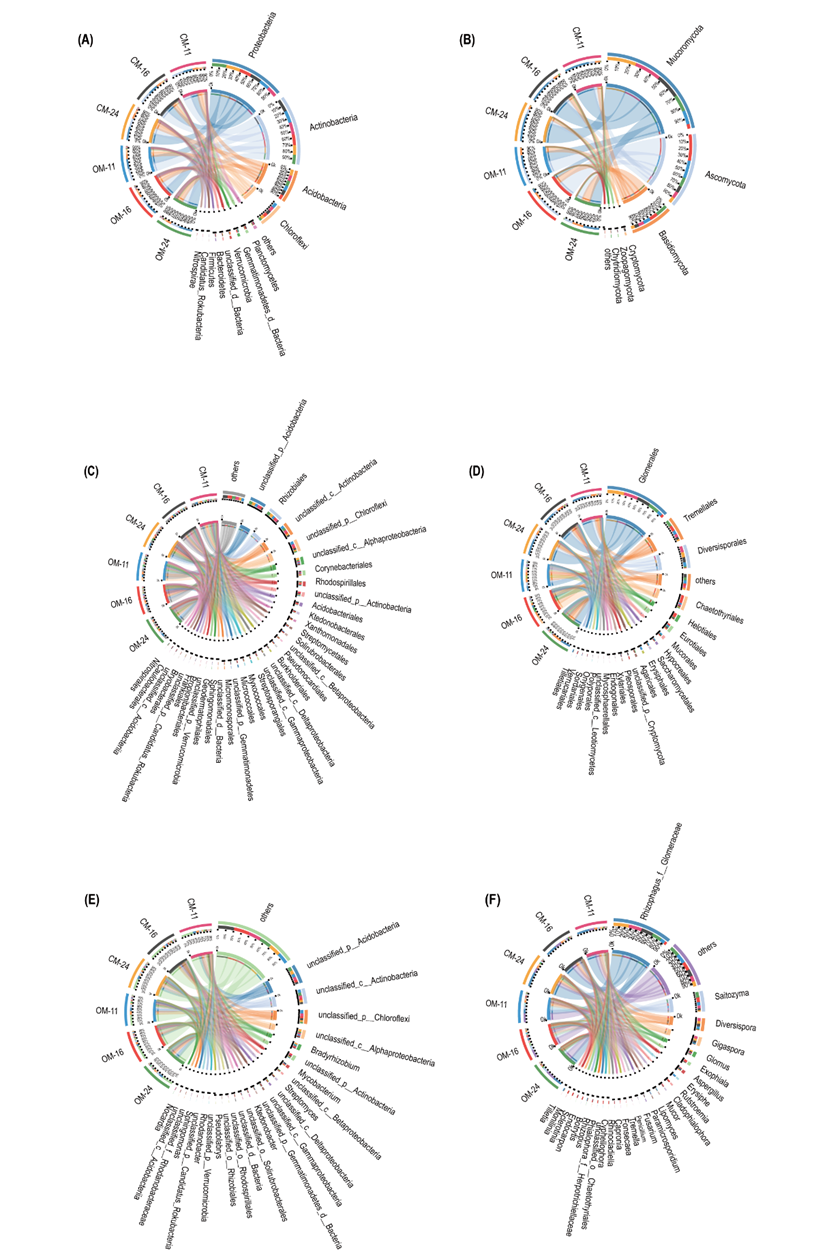


**Figure S1** Composition of bacterial and fungal communities in tea plantation soil under OM and CM (A) Bacterial phylum level (B) Fungal phylum level (C) Bacterial order level (D) Fungal order level (E) Bacterial genus level (F) Fungal genus level. OM represents organic management, CM represents conventional management
